# Meta-Analysis of APP Expression Modulated by SARS-CoV-2 Infection via the ACE2 Receptor

**DOI:** 10.3390/ijms23031182

**Published:** 2022-01-21

**Authors:** Alyssa Caradonna, Tanvi Patel, Matea Toleska, Sedra Alabed, Sulie L. Chang

**Affiliations:** 1 Department of Biological Sciences, Seton Hall University, South Orange, NJ 07079, USA; Caradonna.alyssa@gmail.com (A.C.); Patelta3@shu.edu (T.P.); Toleskam@gmail.com (M.T.); 2 Institute of NeuroImmune Pharmacology, Seton Hall University, South Orange, NJ 07079, USA

**Keywords:** coronavirus-2019, angiotensin-converting enzyme-2, amyloid-beta precursor protein, meta-analysis, severe acute respiratory syndrome-coronavirus-2, Alzheimer’s disease

## Abstract

Alzheimer’s disease (AD) is characterized by the deposition of amyloid-beta (Aβ) plaques from improper amyloid-beta precursor protein (APP) cleavage. Following studies of inflammation caused by coronavirus-2019 (COVID-19) infection, this study investigated the impact of COVID-19 on APP expression. A meta-analysis was conducted utilizing QIAGEN Ingenuity Pathway Analysis (IPA) to examine the link between severe acute respiratory syndrome-coronavirus-2 (SARS-CoV-2) and the modulation of APP expression upon virus binding the Angiotensin-converting enzyme-2 (ACE2) receptor. A Core Analysis was run on the infection by severe acute respiratory syndrome (SARS) coronavirus node, which included molecules affected by SARS-CoV-2, revealing its upstream regulators. Intermediary molecules were found between the upstream regulators and ACE2 and between ACE2 and APP. Activation of the upstream regulators downregulated the expression of ACE2 with a Z-score of −1.719 (*p*-value = 0.086) and upregulated APP with a Z-score of 1.898 (*p*-value = 0.058), showing a less than 10% chance of the results occurring by chance and pointing to an inverse relationship between ACE2 and APP expression. The neuroinflammation signaling pathway was the fifth top canonical pathway involved in APP upregulation. The study results suggest that ACE2 could be downregulated by SARS-CoV-2, resulting in APP upregulation, and potentially exacerbating the onset and progression of AD.

## 1. Introduction

Coronavirus disease-2019 (COVID-19) is caused by the infection of severe acute respiratory syndrome-coronavirus-2 (SARS-CoV-2), whose primary target is the respiratory system. Due to its high transmission rate and risk of fatality, the COVID-19 pandemic has continued to be a large public health concern since 2019. Research is still being conducted to determine the interaction between the spike protein of SARS-CoV-2 and its receptor Angiotensin-converting enzyme-2 (ACE2) on the host cell. ACE2 is a transmembrane protease found mainly in the respiratory epithelia, kidneys, vascular endothelia, and the brain [1]. This protease normally cleaves Angiotensin I and Angiotensin II into Angiotensin 1–9 and Angiotensin 1–7 in relation to the Renin Angiotensin System (RAS) pathway [1]. Upon infection, SARS-CoV-2 interacts with the ACE2 receptor of a host cell via its spike protein S1 subunit, which contains the receptor-binding domain (RBD) [2]. This domain interacts with the ACE2 peptidase domain (PD) to enter the host cell [2]. In severe acute respiratory syndrome-coronavirus (SARS-CoV-1), viral entry occurs through the interaction between the RBD of S1 and the PD of ACE2, which triggers the cleavage of ACE2 by the transmembrane protease serine protease (TMPRSS) 2 and the disintegrin and metallopeptidase domain 17 (ADAM17) [2]. It is hypothesized that SARS-CoV-2 competes with Angiotensin II for binding with ACE2, causing ACE2 to be internalized upon viral entry, reducing and disrupting the RAS pathway [3,4]. With the ambiguity surrounding the molecular internalization following COVID-19 infection and its associated long-term impact on various parts of the body, this study aimed to elucidate the interaction between ACE2 and SARS-CoV-2.

Moreover, new research has shown that following SARS-CoV-2 infection, damage to other organ systems, including the brain, has been detected [3]. COVID-19 infection was found to potentially cause an increase in cognitive dysfunction, directly or indirectly [5]. For instance, post-mortem studies have shown ischemic cerebral white matter damage related to cognitive function [3]. Another study conducted RNA-seq analysis of COVID-19 patients and found a significant increase in amyloid-beta precursor protein (APP) expression compared to non-infected patients [6,7]. The cleavage of APP has been characterized to be one of the hallmarks of Alzheimer’s disease (AD). AD is a neurodegenerative disease, and the most common form of dementia, characterized by a decline in cognitive ability. Normally, the enzymes α-secretase and γ-secretase cleave APP to produce multiple peptides that are involved in axonogenesis, neuronal adhesion, and neurite growth [8]. In AD patients, APP is cleaved by the β-secretase enzyme instead, causing the formation and buildup of amyloid-beta (Aβ) [8]. These increased levels of Aβ cause the production of neurotoxic oligomers, which accumulate and form plaques in the central nervous system (CNS), blocking synapse cell-to-cell signaling [8]. Cytotoxic peptides can also be produced from APP by protease cleavage [9]. These peptides ultimately contribute to the neuronal damage induced by Aβ, increasing synaptic injury [9]. Moreover, another feature of AD brains has recently been found to upregulate ACE2 expression [10,11]. However, in post-mortem human brain tissue, ACE2 levels have been found to be decreased in relation to Aβ [12]. This study also aimed to investigate the consequent impact on amyloid-beta precursor protein (APP) expression following ACE2 modulation by SARS-CoV-2.

At present, dementia and AD have been identified as age-independent comorbidities that increase the risk of COVID-19 related hospitalization and death [13]. In response to the health crisis caused by COVID-19 worldwide, this study seeks to investigate the impact of SARS-CoV-2 upstream regulators on ACE2 and the consequent change in APP expression. An in silico approach was used to conduct a network meta-analysis using QIAGEN Ingenuity Pathway Analysis (IPA) to investigate the possible molecular mechanisms underlying the relationship between SARS-CoV-2 infection and ACE2 expression, and how APP expression is influenced by this interaction. IPA’s Core Analysis tool was used to identify the upstream regulators of the infection by the SARS coronavirus node, which included SARS-CoV-2 affected molecules. The Pathway Explorer tool was then used to identify and connect intermediary molecules between the upstream regulators and ACE2 and between ACE2 and APP. The Molecule Activity Predictor (MAP) tool was used to simulate the exposure of SARS-CoV-2 by activating the upstream regulators in the network, which identified the consequent downstream effects on ACE2 and APP. A Core Analysis was run on the dataset of intermediate molecules, revealing the hepatic fibrosis/hepatic stellate cell activation pathway and the neuroinflammation signaling pathway among the top five canonical pathways. By employing various tools of IPA, our network meta-analyses suggest the downregulation of ACE2 expression following SARS-CoV-2 infection and the consequent upregulation of APP expression as a result of the downregulation of ACE2.

## 2. Results

### 2.1. SARS-CoV Upstream Regulators

To determine the upstream regulators of the SARS-CoV-2 infection, a pathway was created on IPA with a node representing the infection by SARS coronavirus. This node included SARS-CoV-2 and was expanded using IPA’s Grow tool to reveal all the molecules associated with SARS-CoV-2. A total of 679 molecules were found to be associated with this disease. Subsequently, a dataset composed of these 679 molecules was created, and a Core Analysis was run. Chemicals, drugs, and toxicants were excluded prior to analysis as they do not naturally occur in a biological system. The Upstream Analysis feature revealed all the upstream regulators of the SARS-CoV-2 disease, from which the top 20 regulators, as shown in Table 1, were chosen to explore the effects of the disease on ACE2 and APP. The final analysis included 12 of these upstream regulators: interferon gamma (IFNG), tumor necrosis factor (TNF), interleukin 1 beta (IL1B), immunoglobulin, signal transducer and activator of transcription 3 (STAT3), interleukin 6 (IL6), interleukin 10 (IL10), toll-like receptor 3 (TLR3), interleukin 4 (IL4), transforming growth factor 1 (TGFB1), interleukin 1 (IL1), and CD 40 ligand (CD40LG). APP was removed as the later part of the study includes APP as the terminal downstream node in the network. Interferon alpha 2 (IFNA2) and interferon beta (IFN Beta) were removed as they are type I interferons, and their expression by SARS-CoV-2 infection is not fully known according to literature [14]. The remaining six molecules, signal transducer and activator of transcription 1 (STAT1), Interferon alpha, interferon regulatory factor 1 (IRF1), interleukin 2 (IL2), non-POU domain containing octamer binding (NONO), and interferon regulatory factor 7 (IRF7) were removed as they displayed no predicted effects on ACE2 or APP, as revealed by the MAP activation of the pathways.

### 2.2. Positive Impact of Upstream Regulators on APP

**Figure 1:**
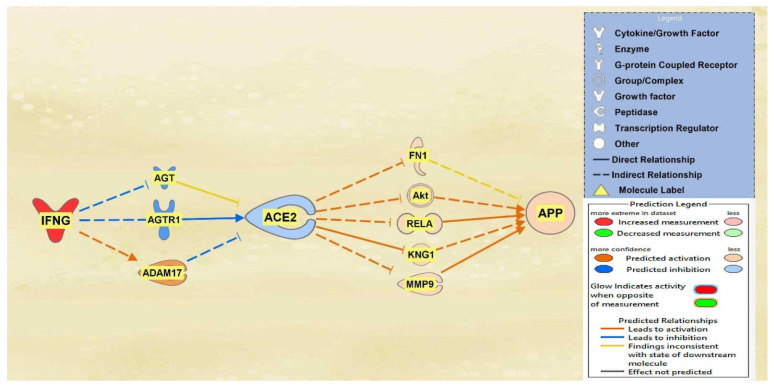
Impact of IFNG on ACE2 and APP. When IFNG is upregulated, ACE2 is predicted to be inhibited. The inhibition of ACE2 led to the overall activation of APP.

IFNG, TNF, CD40LG, the interleukins, and immunoglobulin were all found to overall downregulate ACE2 and subsequently upregulate APP downstream. A total of 10 intermediary molecules were found between IFNG and ACE2 using the Path Explorer tool, excluding chemicals, drugs, toxicants, and any of the 12 upstream regulators. The Path Explorer tool was also used to find a total of 22 intermediary molecules between ACE2 and APP, excluding chemicals, drugs, toxicants, and any of the 12 upstream regulators. The MAP tool was then used to manipulate the pathway indicative of the COVID-19 infection by upregulating IFNG. A total of 7 molecules between IFNG and ACE2 and 17 molecules between ACE2 and APP were removed as they had undetermined expressional changes on the regulation of ACE2 or APP. The intermediary molecules removed between IFNG and ACE2 are listed in Table S1 of the Supplementary Information (SI) document. The intermediary molecules removed between ACE2 and APP are listed in Table S9 of the SI document. The upregulation of IFNG led to the downregulation of angiotensinogen (AGT) and Angiotensin II receptor type 1 (AGTR1) and the upregulation of ADAM17 for an overall downregulation of ACE2. This downregulation of ACE2 led to the upregulation of fibronectin 1 (FN1), Akt, RELA proto-oncogene (RELA), kininogen 1 (KNG1), and matrix metallopeptidase 9 (MMP9) which consequently upregulated APP overall as seen in Figure 1.

**Figure 2:**
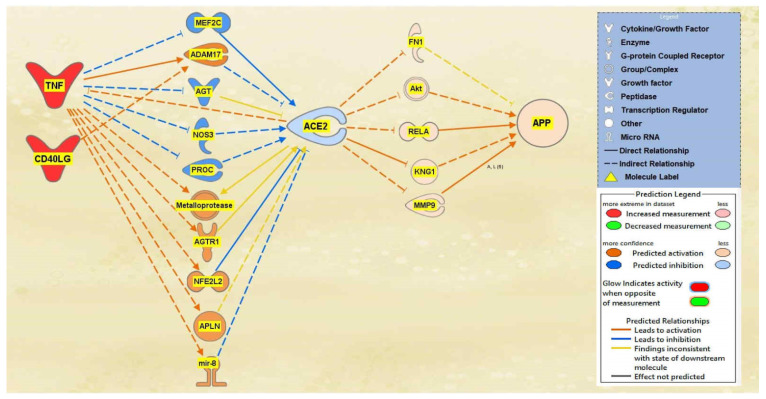
Impact of TNF and CD40LG on ACE2 and APP. When TNF and CD40LG were upregulated, ACE2 was predicted to be inhibited. This inhibition of ACE2 led to the predicted overall activation of APP downstream.

A total of 29 intermediate molecules that show upstream influence towards ACE2 were found between the TNF and CD40LG group and ACE2 using IPA’s Path Explorer tool. Next, chemicals, drugs, toxicants, and any of the 12 upstream regulators were filtered out from the pathway, removing a total of three molecules. APP was then added to the pathway and the Path Explorer tool revealed 22 intermediary molecules between ACE2 and APP. The MAP tool was then used to activate the upstream regulators TNF and CD40LG to predict the effects that SARS-CoV-2 would have on ACE2 and consequently APP. From the 26 upstream biological molecules between TNF and CD40LG and ACE2, 10 remained after MAP activation revealed the intermediary molecules affected by the activation of the upstream regulators. Similarly, five molecules remained between ACE2 and APP, providing the final pathway. The intermediary molecules removed between the TNF and CD40LG group and ACE2 are listed in Table S2 of the SI document. The intermediary molecules removed between ACE2 and APP are listed in Table S9 of the SI document. As shown in the pathway displayed in Figure 2, the activation of TNF and CD40LG downregulated ACE2 and upregulated APP expression.

**Figure 3:**
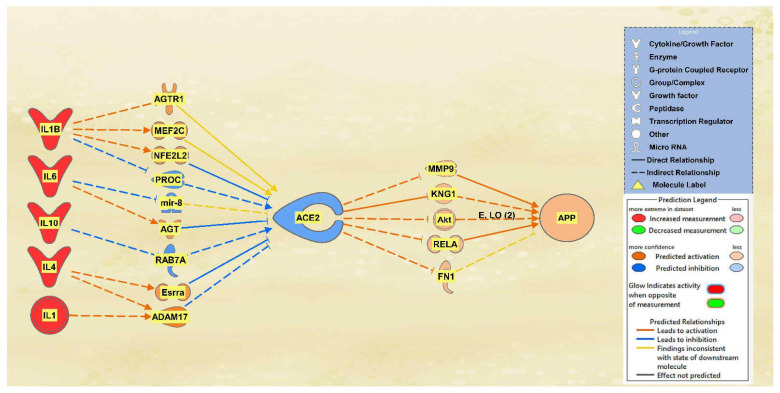
Impact of IL1B, IL6, IL10, IL4, and IL1 on ACE2 and APP. When IL1B, IL6, IL10, IL4, and IL1 were upregulated, ACE2 was predicted to be inhibited. This inhibition of ACE2 led to the predicted overall activation of APP downstream.

A total of 25 intermediate molecules were found between the interleukins IL1, IL10, IL1B, IL2, IL4, and IL6 and ACE2 excluding chemicals, drugs, toxicants, and any of the 12 upstream regulators. Following MAP activation, IL2 was removed for having no predicted effects on the intermediary molecules. A total of 9 of the original 25 intermediates between the interleukins and ACE2 remained, as 16 molecules were removed from the pathway for having no predicted effects on ACE2 after MAP activation. The intermediary molecules removed between the interleukins and ACE2 are listed in Table S3 of the SI document, including IL2, which was removed as well. Following filtration and MAP activation, the same five intermediate molecules remained between ACE2 and APP: FN1, Akt, RELA, KNG1, and MMP9. The intermediary molecules removed between ACE2 and APP are listed in Table S9 of the SI document. The activation of the interleukins group resulted in an overall downregulation of ACE2 and an upregulation of APP. The results are seen in the pathway denoted in Figure 3.

**Figure 4:**
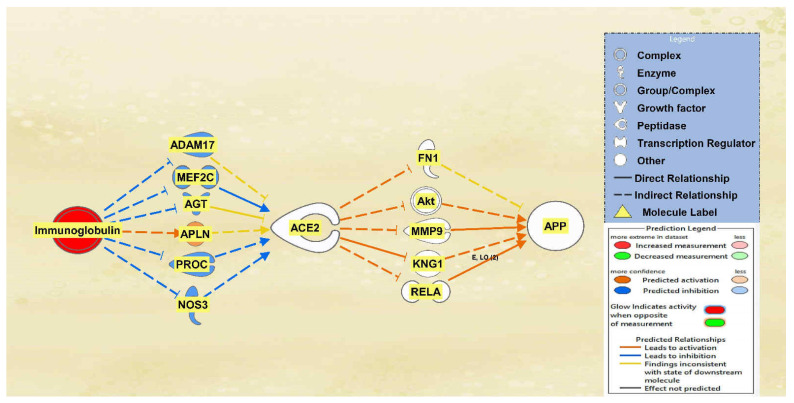
Impact of Immunoglobulin on ACE2 and APP. When immunoglobulin is upregulated, ACE2 is predicted to be inhibited. This inhibition of ACE2 led to the overall very slight activation of APP.

There was a total of 10 intermediates found between immunoglobulin and ACE2 and a total of 22 intermediates found between ACE2 and APP using the Path Explorer tool, excluding all chemicals, drugs, toxicants, and any of the 12 upstream regulators. The MAP tool was then used to manipulate the pathway to mimic the COVID-19 infection by upregulating immunoglobulin. Six intermediary molecules remained between immunoglobulin and ACE2 as four were removed due to not having a downstream effect on ACE2. The intermediary molecules removed between immunoglobulin and ACE2 are listed in Table S4 of the SI document. Immunoglobulin was found to downregulate ADAM17, myocyte enhancer factor 2C (MEF2C), AGT, protein C (PROC), and nitric oxide synthase 3 (NOS3) and upregulate apelin (APLN), leading to the overall less confident downregulation of ACE2 as seen by the light blue color. A total of 17 intermediary molecules were removed between ACE2 and APP as they did not have a downstream effect on APP. The intermediary molecules removed between ACE2 and APP are listed in Table S9 of the SI document. This left a total of five intermediary molecules between ACE2 and APP. The less confident downregulation of ACE2 led to the upregulation of FN1, Akt, MMP9, KNG1, and RELA for an overall very slight upregulation of APP. The results are seen in the pathway denoted in Figure 4.

### 2.3. Negative Impact of SARS-CoV Upstream Regulators on APP

**Figure 5:**
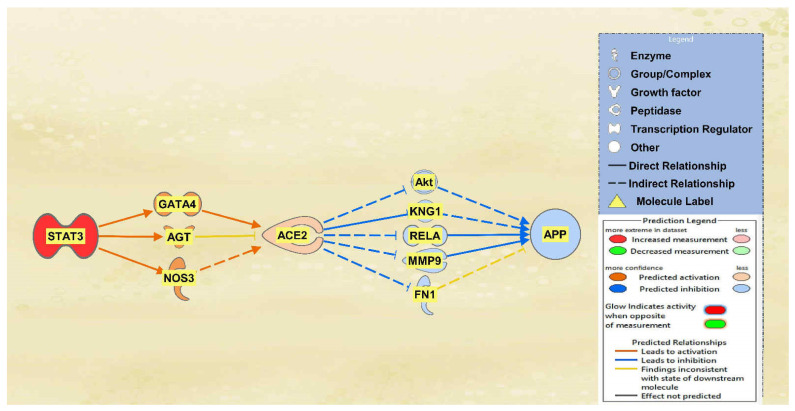
Impact of STAT3 on ACE2 and APP. The upregulation of STAT3 led to the predicted activation of ACE2. This activation of ACE2 led to the overall inhibition of APP.

STAT3, TLR3, and TGFB1 were all found to overall upregulate ACE2 and therefore downregulate APP downstream. A total of 12 intermediary molecules were found between STAT3 and ACE2 while a total of 22 intermediary molecules were found between ACE2 and APP, excluding any chemicals, drugs, toxicants, and any of the 12 upstream regulators. The MAP tool was then used to manipulate the pathway by activating STAT3, as seen in red in Figure 5, to mimic the COVID-19 infection. Nine intermediates were removed between STAT3 and ACE2 as they were not found to have a downstream effect on the regulation of ACE2. The intermediary molecules removed between STAT3 and ACE2 are listed in Table S5 of the SI document. This left three intermediary relationships between STAT3 and ACE2. STAT3 upregulated GATA binding protein 4 (GATA4), AGT, and NOS3, which led to the overall upregulation of ACE2. The predicted activation of ACE2 was less confident as seen by the lighter orange shading due to the inconsistent findings from AGT. There were 17 molecules removed between ACE2 and APP as they did not have an effect downstream on APP, leaving 5 intermediary relationships. The intermediary molecules removed between ACE2 and APP are listed in Table S9 of the SI document. The upregulation of ACE2 led to the downregulation of Akt, KNG1, RELA, MMP9, and FN1, ultimately leading to the downregulation of APP. These predicted inhibitions were also less confident based on the regulation of ACE2 upstream.

**Figure 6:**
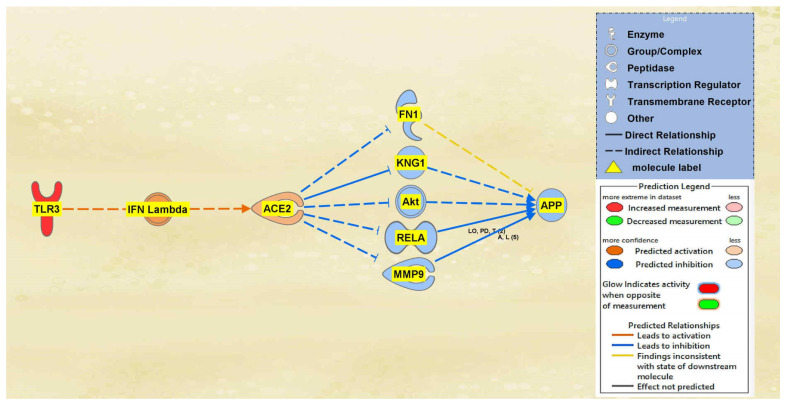
Impact of TLR3 on ACE2 and APP. The upregulation of TLR3 led to the predicted activation of ACE2. The activation of ACE2 led to the overall predicted inhibition of APP.

Between TLR3 and ACE2 a total of 2 intermediary molecules were initially found whereas between ACE2 and APP a total of 22 intermediary molecules were initially found. The intermediaries found excluded chemicals, drugs, toxicants, and any of the original 12 upstream regulators. As seen in Figure 6, the MAP tool was used to manipulate the pathway by activating the TLR3 molecule to simulate the COVID-19 infection. The MAP tool showcased the downstream effects that the upstream regulator, TLR3, and intermediaries have on the regulation of ACE2. One intermediary was removed from the pathway between TLR3 and ACE2 as it did not have any downstream effects on the regulation of ACE2. The intermediary molecule removed between TLR3 and ACE2 is listed in Table S6 of the SI document. This then left one intermediary relationship between TLR3 and ACE2. The only intermediary left is IFN Lambda, which is upregulated by TLR3 contributing to the overall upregulation of ACE2 as well. The predicted activation of ACE2 was less confident as indicated by the slightly lighter orange shading of the node. Seventeen intermediates were removed between ACE2 and APP as they did not have any downstream effects on the regulation of APP, leaving five intermediaries between ACE2 and APP. The intermediary molecules removed between ACE2 and APP are listed in Table S9 of the SI document. The upregulation of ACE2 led to the downregulation of the five intermediaries, FN1, KNG1, Akt, RELA, and MMP9, ultimately leading to the overall downregulation of APP. The predicted inhibition of the five intermediaries and APP were also less confident based on the regulation of ACE2 as indicated by the slightly lighter colored blue of the nodes in Figure 6.

**Figure 7:**
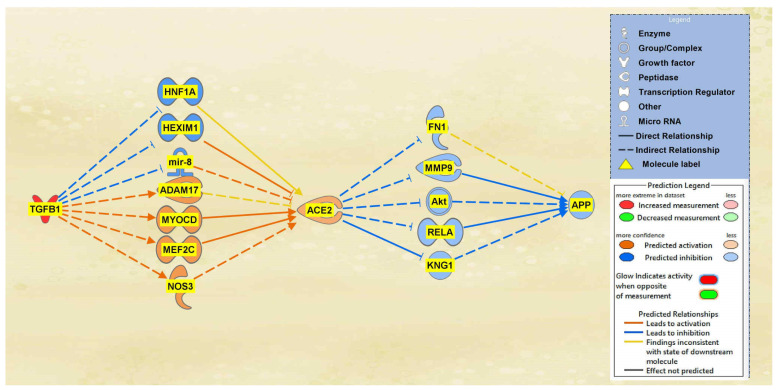
Impact of TGFB1 on ACE2 and APP. The upregulation of TGFB1 led to an overall predicted activation of ACE2. The activation of ACE2 led to the overall predicted inhibition of APP.

A total of 19 intermediaries were found between TGFB1 and ACE2 initially, whereas a total of 22 intermediary molecules were found between ACE2 and APP initially. The intermediaries found excluded chemicals, drugs, toxicants. After the exclusions, as seen in Figure 7, the MAP tool was used to manipulate the pathway by activating the TGFB1 molecule to simulate the COVID-19 infection. The MAP tool showcased the downstream effects that the upstream regulator, TGFB1, and intermediaries have on ACE2. 12 intermediaries were removed from the pathway between TGFB1 and ACE2 as they did not have any downstream effects on the regulation of ACE2, leaving seven intermediary molecules that did show downstream effects on ACE2. The intermediary molecules removed between TGFB1 and ACE2 are listed in Table S7 of the SI document. Activating TGFB1 downregulated HNF1 homeobox A (HNF1A), HEXIM P-TEFb complex subunit 1 (HEXIM1), and microRNA 200c (mir-8); however, activating TGFB1 upregulated ADAM17, myocardin (MYOCD), MEF2C, and NOS3. Overall, these effects contributed to the upregulation of the ACE2 molecule. As shown in Figure 7, the predicted activation of ACE2 was less confident as indicated by the slightly lighter orange shading of the node due to the inconsistent findings of ADAM17 and HNF1A. A total of 17 intermediaries were removed between ACE2 and APP as they did not have any downstream effects on the regulation of APP, leaving 5 intermediaries between ACE2 and APP. The intermediary molecules removed between ACE2 and APP are listed in Table S9 of the SI document. The upregulation of ACE2 led to the downregulation of the five intermediaries, FN1, KNG1, Akt, RELA, and MMP9, ultimately leading to the overall downregulation of APP. The predicted inhibition of the five intermediaries and APP were also less confident based on the regulation of ACE2 as indicated by the slightly lighter colored blue of the nodes in Figure 7.

### 2.4. Quantitative Impact of SARS-CoV Upstream Regulators on ACE2 and APP

**Figure 8:**
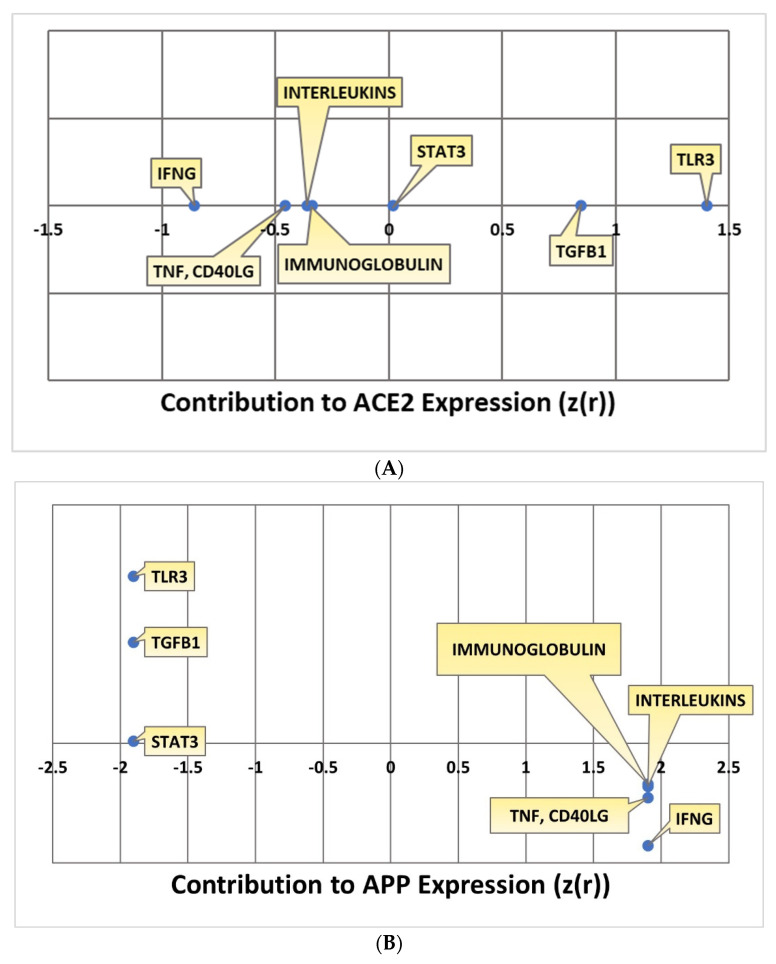
(**A**) Kramer values for ACE2 expression. This chart depicts the Kramer values calculated between each upstream regulator and ACE2. (**B**) Kramer values for contribution to APP expressionfor each upstream regulator.

Kramer’s Downstream Effect analysis is an algorithm that can be used to determine the consistency and statistical likelihood of the observed directionality of the intermediary molecules overall to APP in terms of known literature. Kramer analysis was conducted for each individual pathway. The IFNG, TNF and CD40LG, interleukins, and immunoglobulin pathways all downregulated ACE2. The extent to which ACE2 was impacted by each individual upstream regulator was quantified with a Z-score. IFNG downregulated ACE2 with a Z-score of −0.85506; TNF and CD40LG impacted ACE2 with a –0.4555 Z-score; the interleukins pathway impacted ACE2 with an overall Z-score of -0.36159; the immunoglobulin pathway had an overall Z-score of –0.33982 in terms of its ACE2 downregulation. The STAT3, TLR3, and TGFB1 pathways all upregulated ACE2 with positive Z-scores indicating the extent of the upregulation of ACE2. The STAT3 pathway had an overall Z-score of 0.017867; the TLR3 pathway had an overall Z-score of 1.4; the TGFB1 pathway had an overall Z-score of 0.847524. All the overall Z-scores for ACE2 expression are compiled in Figure 8A. As IFNG, TNF and CD40LG, interleukins, and immunoglobulin all downregulated ACE2, this consequently upregulated APP with a Z-score of 1.902. The upregulation of ACE2 by STAT3, TLR3, and TGFB1 led to the downregulation of APP with a Z-score of −1.90203. All the overall Z-scores for APP expression are compiled in Figure 8B.

### 2.5. Overall Impact of Upstream Regulators on ACE2 and APP

**Figure 9:**
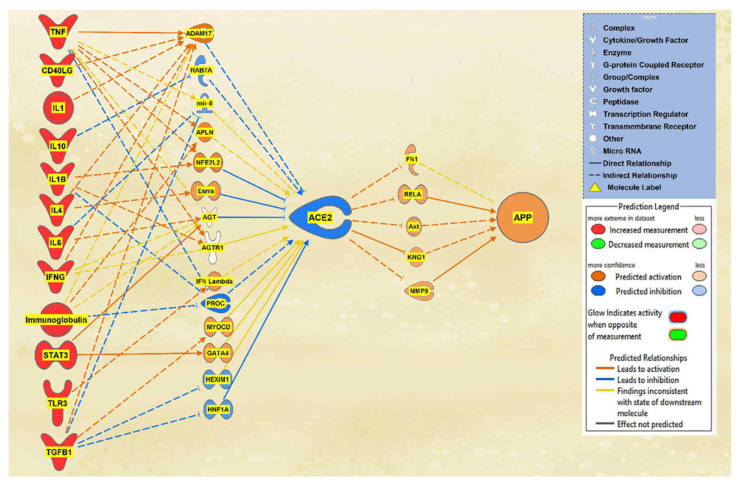
Holistic impact of upstream regulators on ACE2 and APP. This pathway depicts the upregulation of all the upstream regulators leading to the predicted inhibition of ACE2. This inhibition of ACE2 led to the predicted overall activation of APP downstream.

Finally, to observe the overall impact of SARS-CoV-2 on ACE2 and APP, the remaining 12 upstream regulators were added to a pathway. Using the Path Explorer tool to identify the shortest paths, 36 intermediates were found between the upstream regulators and ACE2, and 22 intermediates were found between ACE2 and APP both excluding chemicals, drugs, toxicants, and any of the 12 upstream regulator molecules. The MAP tool was used to manipulate the pathway to imitate the COVID-19 infection by upregulating the upstream regulators. There were 14 intermediaries remaining between the upstream regulators and ACE2 and 5 intermediaries between ACE2 and APP after removing any molecules that did not affect the regulation of ACE2 or APP. The intermediary molecules removed between all the upstream regulators and ACE2 are listed in Table S8 of the SI document. The intermediary molecules removed between ACE2 and APP are listed in Table S9 of the SI document. The overall regulation by the upstream regulators led to a strong downregulation of ACE2 and a strong upregulation of APP which can be seen in Figure 9.

The overall Z-score of the molecules between all the upstream regulators and ACE2 was −1.719, which corresponds to a p-value of 0.085614 on a two-tailed hypothesis test at a significance level of 0.1. This result is significant, and it can be concluded that there is a more than 90% chance that similar results seen will likely not occur due to chance. The overall Z-score of the molecules between ACE2 and APP was 1.898, which corresponds to a *p*-value of 0.57696 on a two-tailed hypothesis test at a significance level of 0.1. This result is significant, and it can be concluded that similar results seen will likely be due to chance.

### 2.6. Canonical Pathways

**Figure 10:**
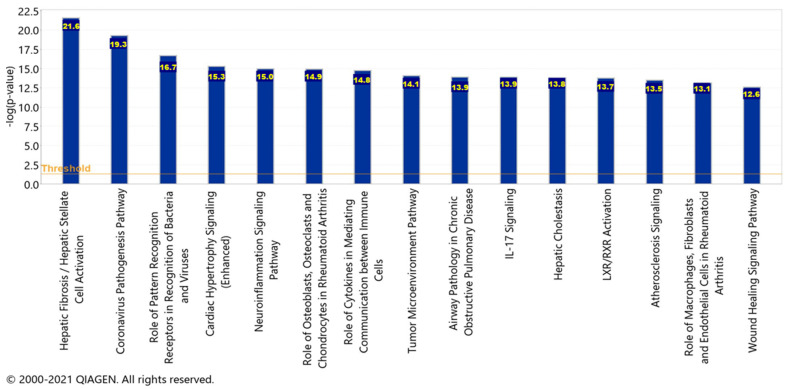
The top 15 canonical pathways. The top 15 canonical pathways and their −log(*p*-value) values related to the pathways represent the overall impact of the upstream regulators.

To acquire the canonical pathways, a Core Analysis was run on the dataset of molecules from the pathway seen in Figure 9, representing the overall impact of the upstream regulators. Chemicals, drugs, and toxicants were filtered out of the Core Analysis. There was a total of 33 molecules in the dataset, however, only 29 of the molecules were mapped IDs (analysis-ready) and the 4 unmapped IDs were Akt, IFN Lambda, Immunoglobulin, and mir-8. Furthermore, there were a total of 287 canonical pathways; however, only the top 15 are being reported with a −log(*p*-value) greater than 12.6 as displayed in Figure 10. 

The significance of each pathway and their –log(*p*-value) values signify the possibility of an association between the molecules from the dataset with each canonical pathway by random chance alone. APP processing and cleavage are implicated in the hepatic fibrosis/hepatic stellate cell activation pathway, the liver X receptor/retinoid X receptor (LXR/RXR) activation pathway, the neuroinflammation signaling pathway, the interleukin 17 (IL-17) signaling pathway, and the atherosclerosis signaling pathway. Within the top 15 canonical pathways, there were four pathways in which the liver was implicated: hepatic fibrosis/hepatic stellate cell activation, hepatic cholestasis, and LXR/RXR activation. Furthermore, the neuroinflammation signaling pathway contained pro-inflammatory cytokines which were found to be upregulated by the SARS-CoV-2 infection.

## 3. Discussion

The aims of this meta-analysis were to investigate the mechanisms by which the SARS-CoV-2 infection modulates APP expression upon binding to the ACE2 receptor. By analyzing the downstream effects of the SARS-CoV-2 upstream regulators on ACE2 expression, found using the infection by SARS coronavirus node, the results indicated inverse modulation of APP expression. This was elucidated through analyzing the impact of individual groups of the upstream regulators: TNF family, interleukin, interferon, transcription regulator, immunoglobulin, TLR3, and TGFB1. From these categories, the TNF family, interleukin, interferon, and immunoglobulin (Figure 1, Figure 2, Figure 3 and Figure 4) were seen to have a positive impact on APP expression via downregulation of ACE2; Figure 5, Figure 6 and Figure 7 show STAT3 (transcription regulator), TLR3, and TGFB1 to have a negative impact on APP expression via upregulation of ACE2. This highlights the possible underlying inverse relationship between ACE2 and APP, where an increase in ACE2 expression decreases APP, while a decrease in ACE2 increases APP expression. While the mechanism by which SARS-CoV-2 infection downregulates ACE2 expression remains to be fully clarified, one of the proposed mechanisms for SARS-CoV-2 is through TNF-α; the infection induces the shedding of ACE2 via the upregulation of TNF-α-converting-enzyme (TACE) [15,16]. This shedding of ACE2 was not noted in other coronaviruses that utilize ACE2 for viral entry. The SARS-CoV-2 virus enters the host cells following the binding to the ACE2 receptor, therefore, both SARS-CoV-2 and ACE2 are then internalized through endocytosis, leading to the surface ACE2 being downregulated [4]. Additionally, there is a potential relationship between COVID-19 and APP metabolism, which has been revealed by several recent multi-omic sample analyses from COVID-19 patients [7]. The RNA-seq analysis of COVID-19 positive patients’ blood samples in comparison to COVID-19 negative patients’ blood samples in the US exhibited a significant increase in APP transcription [7]. Further, a study on a single-cell RNA-seq from post-mortem brain tissues of COVID-19 patients discovered that APP was one of the most upregulated genes in oligodendrocytes [7]. Moreover, a study that genetically engineered a cell line to express human ACE2 and its variants found that the abundance of ACE2 receptors was a severely limiting factor for the spread of the infection within the body [17]. A pseudovirus expressing an S protein, similar to SARS-CoV-2, was found to decrease the expression of ACE2 in infected lungs of hamsters [18]. As ACE2 expression was found to be upregulated in the brains of AD patients [19,20], it follows that SARS-CoV-2 infection has more available ACE2 receptors to bind and cause severe COVID-19 symptoms or exacerbate AD symptoms. This study offers the potential molecules that could be targeted to inhibit the upregulation of APP following ACE2 downregulation by SARS-CoV-2. Further studies are needed to investigate in depth the mechanism by which SARS-CoV-2 downregulates ACE2 expression, possibly through the molecules identified in this study.

Once contracted, the virus has two phases. In phase 1, the virus propagates at a rapid rate by binding onto readily available ACE2 receptors. In phase 2, ACE2 expression begins to diminish significantly, and an uncontrolled inflammatory immune response is triggered, known as the cytokine storm [21]. The intricate molecular events that lead to hyperinflammation are triggered by the attachment of the SARS-CoV-2 spike glycoprotein with ACE2 as the cellular receptor which contributes to the cytokine storm [22]. The major pro-inflammatory cytokines, TNF, IL6, and IL1B, directly stimulate the expression of beta secretase, inducing Aβ processing, and the resulting deposition of Aβ plaques [23]. Furthermore, the Aβ plaque is yielded when APP is cleaved extracellularly by the beta secretase which leaves a 99-residue C-terminal fragment (C99) remaining membrane bound; subsequently, there is an intramembranous cleavage mediated by the gamma secretase [24]. Figure 3 shows the upstream regulators apart of the “cytokine storm” and their impact on ACE2. It can be seen that these molecules, IL1, IL1β IL4, IL6, and IL10, show a marked decrease in ACE2 expression, with a Z-score of −0.36159. This decrease in ACE2 expression caused a consequent increase in APP expression, which could potentially contribute to the exacerbation of Aβ plaques and AD pathogenesis. Further studies are needed to possibly investigate whether the inhibition of the identified intermediates between ACE2 and APP would block the impact of ACE2.

Figure 10 showed the top 15 canonical pathways, in which the top three implicate the liver: the hepatic fibrosis/hepatic stellate cell activation, hepatic cholestasis, and the LXR/RXR activation pathway. Liver fibrosis is the process in which the liver heals any of its injuries through the depositing of extracellular matrix proteins [25]. These injuries are usually caused by different types of hepatitis and other chronic diseases of the liver [25]. These injuries also promote the upregulation of fibrogenic cytokines which activate hepatic stellate cells to their myofibroblastic state downstream, including TNF, transforming growth factor-beta (TGF-β), and IL1 [25]. Although initially providing treatment to the injury, liver fibrosis can become irreversible if progressed to cirrhosis [25]. Angiotensin II (Ang II) is a profibrotic peptide that plays a major role in tissue injury [26]. Ang II is involved in the classical RAS pathway in which Ang II binds the Ang II type 1 receptor (AT1R) to produce vasoconstriction and fibrotic effects [26]. The RAS pathway also involves a protective arm which involves ACE2. In this protective part of the RAS pathway, ACE2 converts Ang II to Ang 1–7 which later binds the MAS oncogene product downstream [27,28]. This conversion by ACE2 mediates the effects of Ang II by further promoting vasodilation along with anti-fibrotic and anti-proliferative effects [29,30].

Although the organs identified as being the most vulnerable to COVID-19 only include the lungs, heart, esophagus, kidney, bladder, and ileum, there are also studies that explore liver damage caused by the viral infection [31,32,33]. One study examined the livers of two post-mortem COVID-19 patients and found viral spike proteins within the cytoplasm of hepatic cells [33]. This study also detected damage to these hepatocytes from the virus and the ability of the virus to replicate itself within these cells [33]. Out of the total 156 patients studied, 64 patients were found to have abnormal levels of liver enzymes including elevated levels of the alanine aminotransferase which are associated with abnormal liver function and injury [33,34]. ACE2 is the binding site of SARS-CoV-2 which is ultimately downregulated upon interaction with the virus [18,35,36,37]. This downregulation of ACE2 could therefore possibly have detrimental effects on the protective RAS pathway in the liver. The downregulation of ACE2 would no longer lead to the degradation and balanced mediation of Ang II from the classical pathway, leading to further damage of the liver. Moreover, the liver is responsible for metabolic detoxification which includes Aβ metabolism via degradation and the excretion in bile [38]. An altered liver status, which could be possible from the COVID-19 infection, can therefore play a major role in the Aβ concentrations throughout the body. There is a possibility that Aβ brain levels can be effected through the changes in Aβ blood concentration due to alterations of liver function [39]. Hepatocytes contain the low-density lipoprotein receptor-related protein 1 (LRP-1) which assists in the mediation of Aβ circulation concentration [40]. When the liver’s activity is altered during disease, LRP-1 expression is decreased, resulting in an increased expression of circulating Aβ [38]. In a study comparing Aβ concentrations from AD patients and patients that did not have dementia, the levels of liver Aβ concentration were lower in AD patients, while the brain Aβ concentrations were higher in AD patients [41]. This study could potentially verify that the dysregulation of liver function, to clear the Aβ circulating and maintain this equilibrium, could lead to Aβ potentially accumulating in the brain.

The LXR/RXR activation pathway was the 12th canonical pathway from the Core Analysis results. The LXR pathway maintains systemic lipid levels, including cholesterol homeostasis of the brain [42]. When the LXR pathway is activated due to elevated cholesterol levels in the brain, the apolipoprotein ApoE, the E4 isoform of the AD risk factor, and ABCA1 or ABCG1 lipid transporters are all expressed to decrease these levels [42]. Activation of this pathway can also lead to a decrease in the Aβ accumulation and protection from neuroinflammation [43,44]. Studies have determined that when LXR is activated, it inhibits the inflammatory responses and inflammatory gene expression related to AD, due to their capability to functionally inactivate the promoters of those pro-inflammatory genes [45]. The heterodimerization between LXR and RXR is required for this transactivation and anti-inflammatory effect [46]. A deficiency in LXRs and the ABCA1 target have been found to increase the Aβ accumulation [42]. When manipulating our related upstream regulators, TLR3, IL1, and TNF, in the LXR/RXR activation canonical pathway using the MAP tool, this led to the downregulation of both LXR and RXR downstream. As seen in our case, the LXR/RXR pathway is being inhibited which could potentially result in further Aβ accumulation and neuroinflammation. The neuroinflammation signaling pathway was the fifth top canonical pathway in the Core Analysis results. Neuroinflammation is a symptom in AD that is caused by the production of neurotoxic oligomers and plaques forming in response to the accumulation of Aβ and tau proteins [47]. These proteins produce an immune response after binding receptors found in the microglia and astroglia, leading to the production of inflammatory cytokines [48]. The Aβ peptides are a key component of amyloid plaques which are derivatives of APP proteolytic cleavage; therefore, it is suggested that Aβ and APP are contributing factors to the pathogenesis of AD [49]. This ultimately progresses and increases the severity of AD as the inflammation is interfering with the processes of the brain [48]. Based on the results, this could point to a potential increase in not only Aβ accumulation but neuroinflammation as well, which are key characteristics in the progression of AD.

A limitation of this study that requires further research was the exclusion of IFNβ and IFNα-2. When SARS-CoV-2 enters via the ACE2 receptor, pathogen-associated molecular patterns (PAMPs) can be sensed, activating IRF3/7 to induce IFN-1 and pro-inflammatory cytokines. However, SARS-CoV-2 causes delayed IFN-1 response [14]. Two of the key immunological manifestations of severe SARS-CoV-2 infection are reduced or delayed IFN-1 response and the cytokine storm (CS) [14]. However, the reduced or delayed IFN-1 response that is caused by SARS-CoV-2 induces paradoxical hyperinflammation. As a result, the organ degenerative effects are worsened [14]. On the other hand, the antiviral properties of IFNβ were found to be very effective against COVID-19 and are being studied in several clinical trials as a potential therapeutic [50]. Though IFNβ and IFNα-2 were seen as upstream regulators in the Core Analysis, they were excluded due to insufficient information regarding expression changes following SARS-CoV-2 infection.

## 4. Materials and Methods

### 4.1. Ingenuity Pathway Analysis Software

The IPA Analysis Match CL license was purchased from QIAGEN (QIAGEN Inc., Germantown, MD, USA, https://www.qiagenbioinformatics.com/products, last accessed on 15 September 2021). IPA is a bioinformatics tool that was used to analyze data and biological processes using the QIAGEN Knowledge Base (QKB) repository composed of over seven million individually modeled relationships to produce and analyze networks through known metabolic and signaling pathways (Figure 1). Data used for ACE2 and APP analysis in this study were retrieved from QKB from 20 July 2021 to 15 September 2021.

### 4.2. Identification of SARS-CoV Upstream Regulators and Canonical Pathways

IPA’s “Grow” tool was used to identify genes, proteins, complexes, and chemicals associated with infection by SARS coronavirus. The “Trim” tool was used to eliminate chemical drugs and toxicants as they do not occur in biological systems. A dataset was created with the remaining molecules and the expression “Core Analysis” was run on the dataset, revealing the top canonical pathways associated with the molecules in the dataset, as well the upstream regulators. The significance of each canonical pathway and upstream regulator was calculated using a Benjamini–Hochberg Corrected Fisher’s Exact test to generate a −log(*p*-value).

### 4.3. Connectivity and Molecule Activity Predictor (MAP)

The overlapping molecules between the upstream regulators and ACE2, as well as the overlapping molecules between ACE2 and APP, were added through the “Pathway Explorer” tool. The “Trim” tool was used to eliminate chemical drugs and toxicants as they do not occur in biological systems. Then, the MAP tool was used to simulate the activation of the upstream regulators as indicated by the data stored in QKB.

### 4.4. Quantitative Analysis of the Influence of SARS-CoV Upstream Regulators on ACE2 and APP Expression

The “Downstream Effect Analysis” algorithm, identified by Krämer, was used to calculate the quantitative weight for the expression change simulated by the “MAP” tool based on the findings stored in QKB. The algorithm uses QKB references as data points to confirm confidence for the change in APP expression through ACE2 upon activation of infection by SARS coronavirus upstream regulators.

(1) $$s\left(e\right)=\mathrm{sgn}\;(\underset{f\;\epsilon \;F\left(e\right)}{\sum }\tilde{s}\left(f\right))$$

(2) $$w\left(e\right)=\frac{1}{N+1}\;\left|\underset{f\;\epsilon \;F\left(e\right)}{\sum }\tilde{s}\left(f\right)\right|$$

(3) O ˜(r):= {v ϵ R(r)|sR(r,v)≠0 ∧v ϵ D}We define the activation Z-score asz(r)= ∑vϵO˜wR(r,v)sR(r,v)sD(v)(∑vϵO˜[wR(r,v)]2)12

The formula identified by Krämer was utilized to compute a Z-score for each intermediate downstream from the infection by SARS coronavirus upstream regulators and upstream of ACE2, and similarly for the molecules downstream of ACE2 and upstream of APP. The scale of individual expression change caused by a molecule in a given pathway runs between −2 and 2, where −2 indicates a strong inhibitory relationship and 2 indicates a strong activation relationship. Each relationship is composed of at least one edge, e, that is in the middle of the cause-and-effect relationship: in other words, an intermediate. Each edge has findings, *f* ∈ *F*(*e*), stored in QKB, upstream and downstream of it. *s*(*e*) identifies the overall sign of a particular direction from the edge; *w*(*e*) identifies the weight of the edge, ranging from [0,1]. Both formulas use the findings, *f*, plugged in as data points of −1 or 1. S_D_ is the sign of the upstream molecule in a given relationship. *z*(*r*) is then used to compute the individual change in expression of the target molecule in a relationship by combining the values computed for each direction downstream and upstream from a particular edge.

Another formula was used to compute an overall Z-score of a particular network combining all the individual Z-scores of each edge relationship. The formula is outlined by Stouffer and used to compute a Z-score aggregating independently found Z-statistics into a two-tailed standard normal distribution.

(4) $$Z_{w}=\frac{\sum_{i=1}^{k}w_{i}Z_{i}}{\sqrt{\sum_{i=1}^{k}w_{i}^{2}}}$$

*Z_w_* is the overall Z-score of the two-tailed standard normal distribution; *Z_i_* is the individual one-tailed Z-score; *k* is the total number of individual Z-scores in the standard normal distribution.

## Figures and Tables

**Table 1:** List of the top 20 upstream regulators.

Upstream Regulator	Molecule Type	*p*-Value of Overlap
IFNG	cytokine	9.57 × 10^−73^
TNF	cytokine	4.57 × 10^−67^
IL1B	cytokine	8.24 × 10^−65^
Immunoglobulin	complex	2.99 × 10^−57^
STAT1	transcription regulator	6.56 × 10^−55^
Interferon alpha	Group	1.3 × 10^−54^
STAT3	transcription regulator	1.3 × 10^−52^
APP	Other	6.25 × 10^−48^
IL6	cytokine	1.52 × 10^−45^
IRF1	transcription regulator	1.36 × 10^−44^
IL10	cytokine	1.56 × 10^−44^
IL2	cytokine	1.31 × 10^−43^
TLR3	transmembrane receptor	1.36 × 10^−43^
NONO	transcription regulator	2.13 × 10^−42^
IL4	cytokine	6.99 × 10^−42^
TGFB1	growth factor	6.35 × 10^−41^
IL1	Group	4.68 × 10^−40^
CD40LG	cytokine	6.99 × 10^−40^
IFNA2	cytokine	2.64 × 10^−39^
IFN Beta	Group	1.5 × 10^−37^
IRF7	transcription regulator	1.7 × 10^−37^

## Data Availability

Data supporting the reported results can be accessed with a purchased license using IPA by QIAGEN (QIAGEN Inc., Germantown, MD, USA, https://www.qiagenbioinformatics.com/products, las accessed on 15 September 2021.

## Supplementary Materials

The following supporting information can be downloaded at: https://www.mdpi.com/article/10.3390/ijms23031182/s1.
