# Supplementary material for: Meta-Analysis of APP Expression Modulated by SARS-CoV-2 Infection via the ACE2 Receptor
Source: Int J Mol Sci. 2022 Jan 21;23(3):1182. doi: 10.3390/ijms23031182 (PMC8835589; doi:10.3390/ijms23031182)
Supplement: Supplementary file 1 [file ijms-23-01182-s001.zip › ijms-1530622-supplementary.pdf]

Supplementary Information (SI)

This supplementary information (SI) document lists the intermediary molecules removed from Figures 1-7 and Figure 9 after MAP tool manipulation revealed that these molecules did not have any downstream effect on ACE2. As the intermediary molecules between ACE2 and APP were the same for Figures 1-7 and Figure 9, the molecules removed between them have been listed for each figure in Table 9 of this SI.

Table S1. Characterization of the intermediate molecules removed between IFNG and ACE2 in Figure 1. *

Symbol	Entrez Gene Name	Location	Type
CALM1 (includes others)	calmodulin 1	Cytoplasm	other
ITGB1	integrin subunit beta 1	Plasma Membrane	transmembrane receptor
NOS3	nitric oxide synthase 3	Cytoplasm	enzyme
PDX1	pancreatic and duodenal homeobox 1	Nucleus	transcription regulator
SMARCA4	SWI/SNF related, matrix associated, actin dependent regulator of chromatin, subfamily a, member 4	Nucleus	transcription regulator
TGFBR2	transforming growth factor beta receptor 2	Plasma Membrane	kinase
VIM	vimentin	Cytoplasm	other

* The identified intermediates were initially filtered out for chemicals, drugs, toxicants, and any of the 12 upstream regulators. The characterization of these intermediary molecules was performed utilizing *QIAGENs Knowledge Base and Ingenuity Pathway Analysis* (i.e. Entrez Gene Name; Location, Family).

Table S2. Characterization of the intermediate molecules removed between the TNF and CD40LG group and ACE2 in Figure 2. *

Symbol	Entrez Gene Name	Location	Family
ASAH1	N-acylsphingosine amidohydrolase 1	Cytoplasm	enzyme
CALM1 (includes others)	calmodulin 1	Cytoplasm	other
CIT	citron rho-interacting serine/threonine kinase	Cytoplasm	kinase

FOXM1	forkhead box M1	Nucleus	transcription regulator
HNF1A	HNF1 homeobox A	Nucleus	transcription regulator
HRAS	HRas proto-oncogene, GTPase	Plasma Membrane	enzyme
HSPA5	heat shock protein family A (Hsp70) member 5	Cytoplasm	enzyme
IFN Beta	--	Extracellular Space	group
IFNG	interferon gamma	Extracellular Space	cytokine
ITGA2	integrin subunit alpha 2	Plasma Membrane	transmembrane receptor
ITBG1	integrin subunit beta 1	Plasma Membrane	Transmembrane receptor
ITGB5	integrin subunit beta 5	Plasma Membrane	other
MDM2	MDM2 proto-oncogene	Nucleus	transcription regulator
NT5E	5'-nucleotidase ecto	Plasma Membrane	phosphatase
PDX1	pancreatic and duodenal homeobox 1	Nucleus	transcription regulator
PDZK1	PDZ domain containing 1	Plasma Membrane	other
RPL10	ribosomal protein L10	Cytoplasm	translation regulator
TGFBR2	transforming growth factor beta receptor 2	Plasma Membrane	kinase
VIM	vimentin	Cytoplasm	other

* The identified intermediates were initially filtered out for chemicals, drugs, toxicants, and any of the 12 upstream regulators. The characterization of these intermediary molecules was performed utilizing *QIAGENs Knowledge Base and Ingenuity Pathway Analysis* (i.e. Entrez Gene Name; Location, Family).

Table S3. Characterization of the intermediate molecules removed between the interleukins and ACE2 in Figure 3. *

Symbol	Entrez Gene Name	Location	Family
ASAH1	N-acylsphingosine amidohydrolase 1	Cytoplasm	enzyme
CALM1 (includes others)	calmodulin 1	Cytoplasm	other
CIT	citron rho-interacting serine/threonine kinase	Cytoplasm	kinase
FOXM1	forkhead box M1	Nucleus	transcription regulator
HNF1A	HNF1 homeobox A	Nucleus	transcription regulator
HRAS	HRas proto-oncogene, GTPase	Plasma Membrane	enzyme
IL2	interleukin 2	Extracellular Space	cytokine
ITGA2	integrin subunit alpha 2	Plasma Membrane	transmembran e receptor
ITGB1	integrin subunit beta 1	Plasma Membrane	transmembran e receptor
ITGB5	integrin subunit beta 5	Plasma Membrane	other
Metalloprotease	--	Extracellular Space	group
NOS3	nitric oxide synthase 3	Cytoplasm	enzyme
NT5E	5'-nucleotidase ecto	Plasma Membrane	phosphatase
PDX1	pancreatic and duodenal homeobox 1	Nucleus	transcription regulator
RPL10	ribosomal protein L10	Cytoplasm	translation regulator
TGFBR2	transforming growth factor beta receptor 2	Plasma Membrane	kinase

VIM	vimentin	Cytoplasm	other

* The identified intermediates were initially filtered out for chemicals, drugs, toxicants, and any of the 12 upstream regulators. The characterization of these intermediary molecules was performed utilizing *QIAGENs Knowledge Base and Ingenuity Pathway Analysis* (i.e. Entrez Gene Name; Location, Family).

Table S4. Characterization of the intermediate molecules removed between immunoglobulin and ACE2 in Figure 4. *

Symbol	Entrez Gene Name	Location	Type
ITGB1	integrin subunit beta 1	Plasma Membrane	transmembran e receptor
ITGB5	integrin subunit beta 5	Plasma Membrane	other
NT5E	5'-nucleotidase ecto	Plasma membrane	phosphatase
SLC6A19	solute carrier family 6 member 19	Nucleus	transcription regulator

* The identified intermediates were initially filtered out for chemicals, drugs, toxicants, and any of the 12 upstream regulators. The characterization of these intermediary molecules was performed utilizing *QIAGENs Knowledge Base and Ingenuity Pathway Analysis* (i.e. Entrez Gene Name; Location, Family).

Table S5. Characterization of the intermediate molecules removed between STAT3 and ACE2 in Figure 5. *

Symbol	Entrez Gene Name	Location	Type
CIT	citron rho-interacting serine/threonine kinase	Cytoplasm	kinase
HNF1A	HNF1 homeobox A	Nucleus	transcription regulator
ITGB1	integrin subunit beta 1	Plasma Membrane	transmembran e receptor
LEPR	leptin receptor	Plasma Membrane	transmembran e receptor
MYOCD	myocardin	Nucleus	transcription regulator

NFE2L2	nuclear factor, erythroid 2 like 2	Nucleus	transcription regulator
SMARCA4	SWI/SNF related, matrix associated, actin dependent regulator of chromatin, subfamily a, member 4	Nucleus	transcription regulator
TBX5	T-box transcription factor 5	Nucleus	transcription regulator
VIM	vimentin	Cytoplasm	other

* The identified intermediates were initially filtered out for chemicals, drugs, toxicants, and any of the 12 upstream regulators. The characterization of these intermediary molecules was performed utilizing *QIAGENs Knowledge Base and Ingenuity Pathway Analysis* (i.e. Entrez Gene Name; Location, Family).

Table S6. Characterization of the intermediate molecules removed between TLR3 and ACE2 in Figure 6. *

Symbol	Entrez Gene Name	Location	Family
IFN Beta	--	Extracellular Space	Group

* The identified intermediates were initially filtered out for chemicals, drugs, toxicants, and any of the 12 upstream regulators. The characterization of these intermediary molecules was performed utilizing *QIAGENs Knowledge Base and Ingenuity Pathway Analysis* (i.e. Entrez Gene Name; Location, Family).

Table S7. Characterization of the intermediate molecules removed between TGFB1 and ACE2 in Figure 7. *

Symbol	Entrez Gene Name	Location	Family
AGT	angiotensinogen	Extracellular Space	growth factor
APLN	apelin	Extracellular Space	other
CALM1 (includes others)	calmodulin 1	Cytoplasm	other

ITGA2	integrin subunit alpha 2	Plasma Membrane	transmembrane receptor
ITGB1	integrin subunit beta 1	Plasma Membrane	transmembrane receptor
ITGB5	integrin subunit beta 5	Plasma Membrane	other
Metalloprotease	metalloprotease	Extracellular Space	group
NT5E	5’-nucleotidase ecto	Plasma Membrane	phosphatase
RPL10	ribosomal protein L10	Cytoplasm	translation regulator
TGFBR2	transforming growth factor beta receptor 2	Plasma Membrane	kinase
VIM	vimentin	Cytoplasm	other
WNK1	WNK lysine deficient protein kinase 1	Cytoplasm	kinase

* The identified intermediates were initially filtered out for chemicals, drugs, toxicants, and any of the 12 upstream regulators. The characterization of these intermediary molecules was performed utilizing *QIAGENs Knowledge Base and Ingenuity Pathway Analysis* (i.e. Entrez Gene Name; Location, Family).

Table S8. Characterization of the intermediate molecules removed between all the upstream regulators and ACE2 in Figure 9. *

Symbol	Entrez Gene Name	Location	Family
ASAH1	N-acylsphingosine amidohydrolase 1	Cytoplasm	enzyme
CALM1 (includes others)	calmodulin 1	Cytoplasm	other
CIT	citron rho-interacting serine/threonine kinase	Cytoplasm	kinase
FOXM1	forkhead box M1	Nucleus	transcription regulator
IFN Beta	--	Extracellular Space	group

ITGA2	integrin subunit alpha 2	Plasma Membrane	transmembrane receptor
ITGB1	integrin subunit beta 1	Plasma Membrane	transmembrane receptor
ITGB5	integrin subunit beta 5	Plasma Membrane	other
LEPR	leptin receptor	Plasma Membrane	transmembrane receptor
MEF2C	myocyte enhancer factor 2C	Nucleus	transcription regulator
Metalloprotease	metalloprotease	Extracellular Space	group
NOS3	nitric oxide synthase 3	Cytoplasm	enzyme
NT5E	5’-nucleotidase ecto	Plasma Membrane	phosphatase
PDX1	pancreatic and duodenal homeobox 1	Nucleus	transcription regulator
RPL10	ribosomal protein L10	Cytoplasm	translation regulator
SLC6A19	solute carrier family 6 member 19	Plasma Membrane	transporter
SMARCA4	SWI/SNF related, matrix associated, actin dependent regulator of chromatin, subfamily a, member 4	Nucleus	transcription regulator
TBX5	T-box transcription factor 5	Nucleus	transcription regulator
TGFBR2	transforming growth factor beta receptor 2	Plasma Membrane	kinase
TMPRSS2	transmembrane serine protease 2	Plasma Membrane	peptidase
VIM	vimentin	Cytoplasm	other
WNK1	WNK lysine deficient protein kinase 1	Cytoplasm	kinase

* The identified intermediates were initially filtered out for chemicals, drugs, toxicants, and any of the 12 upstream regulators. The characterization of these intermediary molecules was performed utilizing *QIAGENs Knowledge Base and Ingenuity Pathway Analysis* (i.e. Entrez Gene Name; Location, Family).

Table S9. Characterization of the intermediate molecules removed between ACE2 and APP in Figures 1-7 and Figure 9. *

Symbol	Entrez Gene Name	Location	Family
ACE	angiotensin 1 converting enzyme	Plasma Membrane	peptidase
ALB	albumin	Extracellular Space	transporter
CASP3	caspase 3	Cytoplasm	peptidase
CASP8	caspase 8	Nucleus	peptidase
CAT	catalase	Cytoplasm	enzyme
CCN2	cellular communication network factor 2	Extracellular Space	growth factor
Collagen(s)	collagen(s)	Extracellular Space	complex
ERK1/2	--	Cytoplasm	group
HRAS	HRas proto-oncogene, GTPase	Plasma Membrane	enzyme
ITGA2	integrin subunit alpha 2	Plasma Membrane	transmembrane receptor
ITGB1	integrin subunit beta 1	Plasma Membrane	transmembrane receptor
MMP2	matrix metallopeptidase 2	Extracellular Space	peptidase
MYOCD	myocardin	Nucleus	transcription regulator
PDYN	prodynorphin	Extracellular Space	transporter
PTGS2	prostaglandin-endoperoxide synthase 2	Cytoplasm	enzyme

TGFBR2	transforming growth factor beta receptor 2	Plasma Membrane	kinase
VIM	vimentin	Cytoplasm	other

* The identified intermediates were initially filtered out for chemicals, drugs, toxicants, and any of the 12 upstream regulators. The characterization of these intermediary molecules was performed utilizing *QIAGENs Knowledge Base and Ingenuity Pathway Analysis* (i.e. Entrez Gene Name; Location, Family).
